# The Editor's Letter-Box

**Published:** 1916-04-22

**Authors:** 


### THE EDITOR'S LETTER-BOX.

### A Matron's Worries.

*To the Editor of* The Hospital.

Sir,—I note in your last issue the difficulties experienced by "Matron, Military Hospital." I think she would find an oil mop a great assistance to her untrained helpers in finishing the stained floors after they have been swept. We have used them now for some time, and are no longer bothered with floating bits of flue and a dusty appearance. The O-Cedar mop, an American invention, costs, I think, six or seven shillings, and the oil with which it must be replenished every few days is about 4s. a quart. There is a British make of mop with special oil which is rather less expensive, but it lacks the peculiarly refreshing odour of the O-Cedar. I suspect any kind of string mop used with any kind of thin oil would serve the purpose.—Yours truly,

Sister Amice.

